# Right cerebral motor areas that support accurate speech production following damage to cerebellar speech areas

**DOI:** 10.1016/j.nicl.2021.102820

**Published:** 2021-09-20

**Authors:** Sharon Geva, Letitia M. Schneider, Sophie Roberts, Shamima Khan, Andrea Gajardo-Vidal, Diego L. Lorca-Puls, PLORAS team, Thomas M.H. Hope, David W. Green, Cathy J. Price

**Affiliations:** a Wellcome Centre for Human Neuroimaging, Institute of Neurology, University College London, 12 Queen Square, London WC1N 3AR, United Kingdom; b Department of Cognition, Emotion, and Methods in Psychology, Faculty of Psychology, University of Vienna, Universitätsring 1, 1010 Vienna, Austria; c Faculty of Health Sciences, Universidad del Desarrollo, Concepcion, Chile; d Department of Speech, Language and Hearing Sciences, Faculty of Medicine, Universidad de Concepcion, Concepcion, Chile; e Department of Experimental Psychology, Faculty of Brain Sciences, University College London, London, United Kingdom

**Keywords:** Cerebellum, fMRI, Premotor cortex, Supplementary motor area

## Abstract

Specific regions of the cerebellum are activated when neurologically intact adults speak, and cerebellar damage can impair speech production early after stroke, but how the brain supports accurate speech production years after cerebellar damage remains unknown. We investigated this in patients with cerebellar lesions affecting regions that are normally recruited during speech production. Functional MRI activation in these patients, measured during various single word production tasks, was compared to that of neurologically intact controls, and patient controls with lesions that spared the cerebellar speech production regions. Our analyses revealed that, during a range of speech production tasks, patients with damage to cerebellar speech production regions had greater activation in the right dorsal premotor cortex (r-PMd) and right supplementary motor area (r-SMA) compared to neurologically intact controls.

The loci of increased activation in cerebral motor speech areas motivate future studies to delineate the functional contributions of different parts of the speech production network, and test whether non-invasive stimulation to r-PMd and r-SMA facilitates speech recovery after cerebellar stroke.

## Highlights

• Participants with damage to cerebellar speech regions were studied with fMRI.
• At the time of test, their speech production was accurate and precise.
• Their speech production activation was enhanced in right hemisphere motor regions.
• We provide hypotheses for targeting future fMRI and brain stimulation studies.

## Graphical abstract

**Figure:**
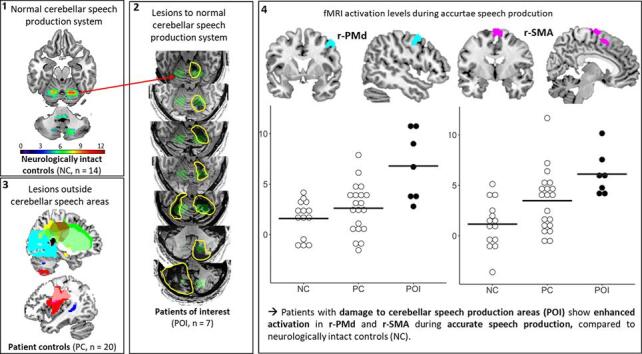

## 1 Introduction

Many prior studies of stroke patients have shown cerebellar involvement in various aspects of motor control, including motor control of speech (i.e. dysarthria). For example, an epidemiological study of cerebellar stroke found that around 40% of patients with cerebellar damage showed signs of dysarthria upon examination early after stroke, suggesting the involvement of the cerebellum in articulation (Tohgi et al., 1993). There is also evidence that cerebellar damage can cause apraxia of speech (De Witte et al., 2017, Mariën et al., 2015), which according to Mariën et al. (2015) might have similar underlying motor planning difficulties as ataxic dysarthria. A key observation, first noted by Holmes (1917) more than a hundred years ago and endorsed by others (Schmahmann and Sherman, 1998), is that a large proportion of patients with cerebellar stroke have speech production impairments that recover completely within the first few months after insult. The goal of our study was to investigate the neural basis of accurate speech production years after stroke in patients with cerebellar damage to known speech production regions.

Prior research with cerebellar patients has yielded inconsistent findings with regard to which cerebellar region is responsible for impaired speech articulation. A voxel-based lesion-symptom mapping study found that dysarthria was most commonly associated with lesions to superior cerebellar lobules IV-VI in a group of acute stroke patients, and with the posterior part of the dentate nucleus, in both acute and chronic patients (Schoch et al., 2006). Damage to the superior cerebellar artery (SCA) territory has also been associated with dysarthric symptoms in other studies (Ackermann et al., 1992, Lechtenberg and Gilman, 1978), with two-thirds of patients with SCA territory lesions diagnosed with dysarthria (Erdemoglu and Duman, 1998). However, a subsequent study reported that only half of patients with dysarthria sustained lesions affecting the SCA. The other half had lesions in the posterior and/or anterior inferior cerebellar artery (PICA/AICA) territory, most commonly to the rostral paravermal region of the anterior lobe (Urban et al., 2003), consistent with a later lesion-symptom mapping study (Byoung et al., 2010). In contrast, no patients with lesions in the territory of the lateral branch of the PICA were diagnosed as having developed dysarthria (Barth et al., 1994).

The extent to which speech impairment is associated with lesions to either cerebellar hemisphere is also unclear. Ackerman et al. (1992) found that among patients with cerebellar damage and speech impairments, lesions were as likely to affect the right as the left cerebellar hemisphere. This contrasts with an earlier finding that two thirds of dysarthric patients with cerebellar damage had exclusively, or predominantly, left cerebellar damage (Lechtenberg and Gilman, 1978) and a later finding that two thirds of dysarthric patients with cerebellar damage had unilateral right hemisphere damage (Urban et al., 2003).

A limitation of some prior lesion studies is that they included patients with large and/or non-isolated lesions. Co-occurrence of damage to multiple regions makes it difficult to dissociate their contributions to the behavioural impairment. For this, we turn to functional imaging studies that have demonstrated cerebellar involvement in motor aspects of speech, in neurologically intact participants and provided a fine-grained anatomical localisation of the speech production regions within the cerebellum.

The most consistent finding from these fMRI studies is of activation in bilateral cerebellar lobule VI during tasks requiring articulation, such as voiced speech over whispered speech (Correia et al., 2020), pseudoword reading over silent viewing of meaningless strings (Peeva et al., 2010), mouthing over inner speech (Nota and Honda, 2004), articulating single simple vowels (Grabski et al., 2012), pseudoword repetition (Rauschecker et al., 2008), and when using various tasks that manipulated syllable and sequence complexity (Bohland and Guenther, 2006). The association of bilateral cerebellar lobule VI with speech articulation is also supported by evidence showing that the tongue representation in cerebellar lobule VI (mapped using a motor task) is functionally connected to the contralateral somatomotor cerebral cortical regions that control tongue movements (Buckner et al., 2011). A further demonstration of the consistency of lobule VI activation comes from Callan et al. (2007), who reviewed studies of cerebellar involvement in speech production tasks (e.g. syllable repetition or saying the months of the year) and found that activation peaks in bilateral lobule VI were reported in all studies. In contrast, an earlier study by Shuster and Lemieux (2005) found activation only in right lobule VI during repetition of multisyllabic over monosyllabic words.

Activation in lobules VIIIa and VII/Crus I has also been related to speech production. In lobule VIIIa, activation has been reported bilaterally (Bohland and Guenther, 2006, Brown et al., 2009, Nota and Honda, 2004), but Callan et al. (2007) found that only 1/8 of the reviewed studies reported bilateral lobule VIIIa activation, and Nota and Honda (2004) associated the lateral portions of bilateral lobule VIIIa with articulation, but more medial parts with vocalisation without articulation (i.e. voicing aahhh). In lobule VII/Crus I, activation was reported bilaterally (Bohland and Guenther, 2006, Ghosh et al., 2008, SHUSTER and LEMIEUX, 2005), or only in the right hemisphere (Rauschecker et al., 2008). In other studies, the importance of activation in the most inferior portion of the cerebellum may have been missed due to the limited field of view used during fMRI data acquisition (Brown et al., 2009).

In terms of how these cerebellar regions contribute to speech production, Guell et al. (2018b) suggested that bilateral lobule VI, which shows the most consistent activation across studies, is responsible for motor execution (termed ‘primary motor representation’). This is supported by the findings of Grabski et al. (2012), who demonstrated bilateral lobule VI activation when participants moved their jaw, lips or tongue without articulating. Bilateral lobule VIII, on the other hand, has been described as a ‘secondary motor representation’ which is engaged only in tasks of greater motor complexity or those requiring planning or attention (Guell et al., 2018a, King et al., 2019). In line with this functional distinction, a study reporting bilateral activation in ‘primary motor representation’ and in right ‘secondary motor representation’ during initial pseudoword repetition, showed that a learning effect (i.e. reduced activation in subsequent trials) could only be observed in the area of ‘secondary motor representation’ (Rauschecker et al., 2008).

Given that some parts of the cerebellum are associated with speech production in the intact brain, and that a damaged cerebellum can give rise to speech production impairments, a major question remains: How do patients with cerebellar damage recover speech production over time? To the best of our knowledge, only one study to date examined the neural regions that support recovery after adult cerebellar stroke using fMRI but this was in a hand grasping task (Kinomoto et al., 2003). Specifically, the authors found that six stroke patients with recovered hand grasping ability showed increased activation in the contralateral cerebellar hemisphere and ipsilateral sensorimotor cerebral cortex (relative to the location of the cerebellar lesion). No studies have so far investigated functional reorganization and the neural regions that support accurate speech production following cerebellar stroke.

In the current study, we used fMRI to investigate how patients with isolated lesions to cerebellar speech production regions were able to accurately produce speech years after stroke. We classified whether each cerebellar lesion site overlapped with speech production regions derived from an fMRI study of neurologically intact participants, rather than relying on anatomical boundaries that do not necessarily agree with functional boundaries (King et al., 2019). We defined ‘normal’ cerebellar speech production regions as those areas that were significantly activated by a group of neurologically intact participants during a series of speech production tasks. These tasks required overt production of single words, allowing us to identify regions associated with speech production across stimulus modality (auditory vs. visual), response mode (naming, reading or repetition) and lexicality (words vs. pseudowords) and to assess regional sensitivities to these variables. This was motivated by the fact that such linguistic parameters are known to affect speech production (Forster and Chambers, 1973, Saletta et al., 2016, Vitevitch, 2002).

We hypothesised that, when producing speech, patients with damage to cerebellar speech regions would show higher than normal activation in undamaged areas in the ipsilesional cerebral cortex and/or contra-lesional cerebellum (as documented in Kinomoto et al., 2003). We tested this hypothesis in a study that addressed the following questions: (i) Do patients of interest (with damage to cerebellar speech production regions) show higher than normal activation during speech production tasks? (ii) Is abnormally high activation unique to the patients of interest, or is it also seen following lesions that spare the cerebellar speech production regions? (iii) Can activation in our patients of interest be related to demographic, behavioural, and lesion related variables? and, (iv) Is enhanced activation related to stimulus modality, phonology, semantics or lexicality?

By investigating these questions, we aim to obtain a better understanding of the neural systems that support accurate speech production following damage to cerebellar speech production regions.

## 2 Materials and methods

### 2.1 Participants

Three groups of native English speakers (patients of interest, n = 7; patient controls, n = 20; and neurologically intact controls, n = 14) with normal or corrected-to-normal vision and hearing gave written informed consent according to the Declaration of Helsinki prior to being included in the study. They were compensated financially for their time in accordance with the London Queen Square Research Ethics Committee (study code: 13/LO/1515 and 19/LO/1755).

All patients that completed our fMRI paradigm were initially recruited to the Predicting Language Outcome and Recovery After Stroke (PLORAS) database, which records behavioural, demographic and imaging data from participants with a history of adult stroke, and with no other neurological condition (Seghier et al., 2016). The PLORAS study includes any patient with a stroke that, according to clinical reports, resulted in (i) a lesion >1 cm^3^; and/or (ii) a sensory, motor or cognitive impairment lasting beyond the first week after stroke. All patients are assessed with a standardised language battery (see below for more details), and if their speech production abilities are sufficiently good, and they are able to tolerate the MRI environment, they are invited to functional imaging at a later date.

**Fig. 1:**
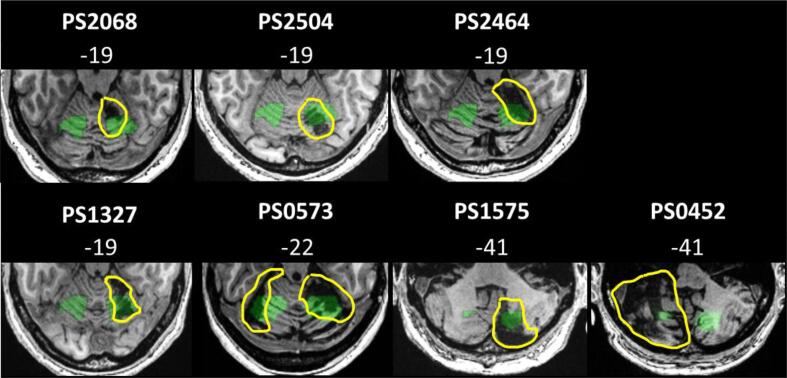
Lesions in patients of interest affecting the cerebellar speech production regions. Axial slices in MNI space of individual T1-weighted images, showing the lesions in the 7 patients of interest. Lesions are highlighted in yellow for better visualisation. Overlaid in green are binarised clusters of activation associated with speech production in neurologically intact controls (n = 14) (activation shown in bilateral cerebellar lobules V/VI, and Crus II/VIIb/VIIIa), derived from the main effect of 8 speech production tasks, with a voxel-level statistical threshold of p < 0.05 FWE-corrected across the whole brain. z coordinates and patients’ ID from the PLORAS database are displayed above each image.

**Fig. 2:**
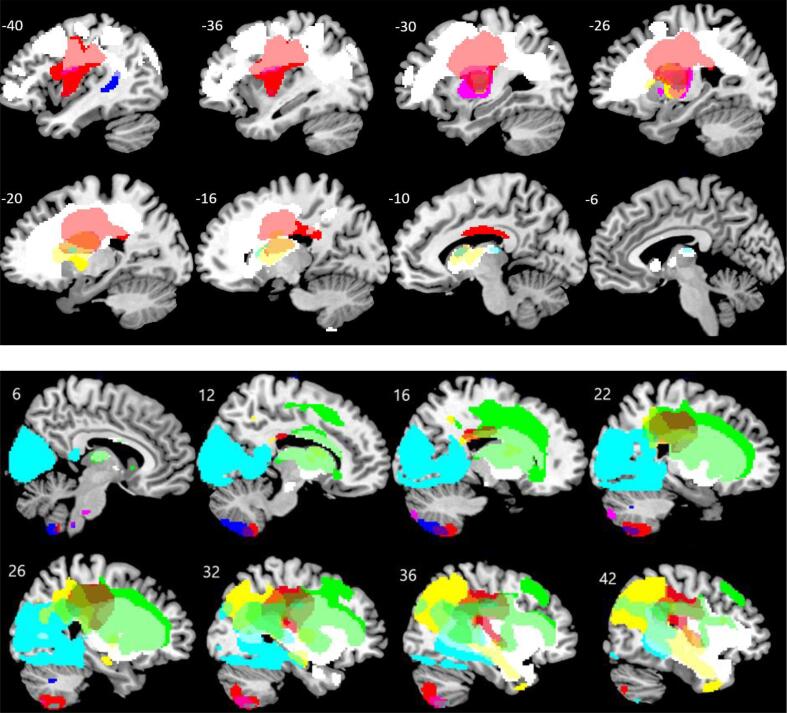
Lesion locations of the patient controls. Binary lesion images are overlaid on sagittal slices in MNI space, each colour represents a lesion of one patient (n = 19, as the lesion for PS0472 was not identified by the automated lesion identification software). × coordinates are displayed above each image. See Supplementary Table 1 for further details of the lesion site in each patient control.

Our ‘patients of interest’ (POI, n = 7; see Fig. 1 and Table 1) all had focal damage to speech production regions that were identified from fMRI data of neurologically intact controls (NC, n = 14; see Fig. 1) with no history of any neurological or psychiatric disorders. All other patients, assessed with the same paradigm and scanner, were assigned to a ‘patient control’ group (PC, n = 20, after excluding 8 patients whose in-scanner accuracy was lower than that of the patients of interest; i.e. < 62.5% on any of the tasks). Hence, patient controls were stroke survivors with lesions that spared the speech production regions in the cerebellum (see Supplementary Table 1 and Fig. 2 for lesion location and other details related to the patient controls).

**Table 1:** Demographics and testing details for each patient of interest.

Patient ID	Lesion location	Age at stroke	Gender	Lesion volume (cm^3^)	Hand	Stroke to CAT (months)	Stroke to fMRI (months)
PS2068	R lobule V, VI	43.6	M	1.4	R	5	42
PS2464	R lobule V, VI	44.0	M	2.0	R	10	35
PS1327	R lobule V, VI	60.8	M	0.5	R	4	61
PS2504	R lobule VI	54.5	M	0.9	R	14	33
PS0573	R lobule VI, Bilateral Crus I	21.8	F	16.2	R	205	289
PS1575	R Crus I, Crus II / lobule VIII	72.0	M	12.3	L	13	71
PS0452	L lobule VIIb, VIII, Crus II	54.2	M	30.1	R	37	133

Hand = dominant hand: Left (L) or Right (R). Gender = Male (M) or Female (F).

Patients of interest did not differ from the other groups in age at test (independent sample *t*-test, p > 0.05), gender (Chi Square test, p > 0.05), or handedness (assessed using the Edinburgh Handedness Inventory (Oldfield, 1971), Chi Square test, p > 0.05). Nor did they differ from the patient control group in age at stroke or time since stroke (independent sample *t*-test, p > 0.05 for both). Lesion volume was smaller for patients of interest than patient controls (independent sample *t*-test, t_(25)_ = 2.89, p = 0.008). The demographic and clinical details for each group of participants are summarised in Table 2, and the potential influence of these and other variables is considered in the Results section.

**Table 2:** Demographic and clinical details of the participant groups.

Variable		POI (n = 7)	PC (n = 20)	NC (n = 14)
Age at fMRI (years)	M (SD)	58 (12.3)	56 (9.5)	44 (19.8)
	Range	46–78	40–73	23–81
Gender, n.	Males	6	14	7
	Females	1	6	7
Handedness, n.	R / L / Ambi	6 / 1 / 0	15 / 4 / 1	12 / 2 / 0
Age at stroke onset (years)	M (SD)	50 (15.8)	49 (9.9)	N/A
	Range	22 – 72	31 – 69	
Stroke to scan (months)	M (SD)	95 (92)	80 (67)	N/A
	Range	33–289	10 – 225	
Lesion volume (cm^3^)*	M (SD)	45.7 (53.4)	9.1 (11.2)	N/A
	Range	0.5–30	0* − 152	

NC = neurologically intact controls, PC = patient controls, POI = patients of interest; M = mean, SD = standard deviation; Handedness: R = right, L = left, Ambi = ambidextrous. *0 = the automated lesion identification software did not segment the lesion (see section 2.5) and therefore the lesion volume is defined as 0 cm^3^.

### 2.2 Assessment of speech abilities

Patients’ speech abilities at the acute stage are described based on medical notes and self-rating. For self-rating, patients retrospectively evaluated their speech abilities at 1 month after their stroke, indicating whether they could produce gestures but not speech, single words, phrases or short sentences, were nearly back to normal or speaking normally.

Patients also completed the Comprehensive Aphasia Test (CAT; Swinburn et al., 2004) at minimum 3 months post-stroke, and before the fMRI session (time between stroke and CAT: average = 41 ± 73.2 months, range: 4.1 – 205.4 months for POI; average = 49 ± 42.8 months, range: 3.7 – 148.5 months for PC). The CAT includes a 6-task cognitive screen and 21 speech and language tests that are administered and scored by registered Speech and Language therapists. We report patients’ speech abilities measured on single word Naming and Repetition tasks, and a Spoken Picture Description task. For Naming, line drawings of 24 objects (e.g. pyramid) and 5 actions (e.g. threading a needle) are presented one at a time, with instructions to name them aloud. In the three Repetition tasks, participants are presented with 16 spoken words, 3 complex words, and 5 non-words, one at a time, for immediate repetition. For Spoken Picture Description, participants are asked to describe a picture that shows a complex scene, and their verbal description is recorded.

The Naming, Repetition and Spoken Picture Description scores reflect whether the patient made any articulatory errors, including apraxic and obvious dysarthric errors, which affect the perceptual identity of the target word. Recognising that the CAT does not penalise mild dysarthric distortion, we also checked the individual responses for each of those subtests for any additional articulation errors that may suggest mild dysarthria.

Individual responses for the fMRI Object Naming and Word Repetition tasks (see details below) were screened for articulation errors in the same manner.

The Spoken Picture Description recordings were also scored using the ‘Inventory of Articulation Characteristics of Apraxia’, taken from the Apraxia Battery for Adults, 2nd edition (Dabul, 1979). The inventory records 15 behaviours associated with speech production, including various types of errors (e.g. phonemic anticipatory, perseverative, transposition, voicing, or vowel errors), visible/audible searching or off-target attempts at the word, difficulty with speech initiation, abnormal prosodic features, awareness of errors and ability to correct them, among others. A score of 5 and above is indicative of apraxia.

### 2.3 fMRI speech production tasks

For each patient, the fMRI study was conducted after the CAT assessment (time between CAT and fMRI: average = 53 ± 28.9 months, range: 18.9 – 95 months for POI; average = 30 ± 36.8 months, range: 1.5 – 115 months for PC).

All participants performed 8 tasks requiring overt production of single words or pseudowords, as previously described by Oberhuber et al. (2016). The tasks factorially varied the demands on phonological processing, semantic processing, and input modality (visual or auditory). In the visual modality (v) the tasks were: Reading aloud written object names (vW), Reading aloud written pseudowords (vP), Naming aloud the object in a picture (vO), and Naming aloud the colour of meaningless patterns (vC). In the auditory modality (a) the corresponding tasks were: Repeating aloud heard words (aW) that were always object names, Repeating aloud heard pseudowords (aP), Naming aloud the object or animal that generated heard sounds (aO), and Naming aloud the gender (male or female) of a person humming without articulating any phonologically recognizable speech sounds (aH). For all participants, the order of conditions was: vW, aW, vO, vC, aO, vP, aP, and aH. The order was kept the same to ensure that inter-subject variability cannot be explained by task order or stimulus effects.

Participants performed 5 additional tasks within the same scanning session, previously described by Sanjuán et al. (2015). These tasks (silent visual and auditory semantic matching, sentence production, verb production and naming two objects) were part of a different experiment, and are not reported here as they were not relevant to our research questions.

#### 2.3.1 Stimulus selection

We selected 120 familiar animals and objects that were easily recognisable from coloured drawings. These were used for the 8 tasks reported here as well as for the 5 tasks not reported here. For the auditory object naming task, 8 objects were added because pilot testing in an independent sample of 8 neurologically intact participants indicated that only a limited number of the objects have easily recognisable sounds. To minimise stimulus repetition across the two paradigms, we alternated the modality in which objects were presented (e.g. naming objects from pictures (vO) presented objects whose names had previously been heard during auditory semantic matching). Any cross-modality priming of repeated object concepts was consistent across all participants.

All sounds for the auditory object naming condition were taken from the NESSTI sound library (Hocking et al., 2013). The non-semantic stimuli were matched to the semantic (object) stimuli as follows: Pseudowords (for reading or repeating) were created using a non-word generator (Duyck et al., 2004) and matched to the real words for bigram frequency, number of orthographic neighbours and spoken word length (for details of characteristics see Oberhuber et al., 2016). The meaningless nonverbal visual stimuli were coloured patterns, created by scrambling both global and local features of the object pictures, and then manually edited to accentuate one of 5 colours (i.e. blue, orange, red, yellow and green). Images judged to have any identifiable features were discarded, and an independent group of 19 neurologically intact participants named the colours of all stimuli to ensure speech production responses were consistent for each colour.

#### 2.3.2 Procedure

Prior to scanning, each participant was trained on all tasks. For 7 of the tasks, we used a set of stimuli different from that presented in the scanner. For naming objects from sounds, we presented the same stimuli during training and scanning because neurologically intact participants required more practice on this condition to achieve highly accurate and consistent object recognition. During fMRI data acquisition, the object sound stimuli were presented in a different order than during the practice session.

Each task was executed during its own scanning run with 4 blocks of 10 trials alternating with 16 s of resting while fixating on a central cross. Scanning started with the instructions “Get ready” displayed on the screen during which 5 dummy scans were collected. Each of the four blocks was preceded by a displayed instruction to prepare for the next condition (e.g. “Repeat”), lasting for the length of one Repetition Time (TR = 3080 ms), and followed by 16 s of rest. Trials were presented at a rate of 1 trial every 2.5 s. Visual stimuli remained on the screen for 1.5 s followed by 1 s fixation. The mean durations for auditory stimuli were: 0.63, 0.65, 1.45 and 1.05 s for words, pseudowords, object sounds and humming, respectively. Data acquisition per participant lasted an average of 90 min including out-of-scanner training, setting up, getting the participant into the scanner, and structural and functional imaging.

Stimuli were presented using COGENT (http://www.vislab.ucl.ac.uk/cogent.php) and run in MATLAB 2010a (MathWorks, Sherbon, MA, USA). During all conditions participants were asked to keep their body and head as still as possible and their eyes open with fixation on the cross at the centre of the screen. Visual stimuli were presented using an LCD projector on a screen placed at the head-end of the scanner bore and an adjustable mirror placed on the head coil to allow participants’ viewing of the screen. Pictures subtended a visual angle of 7.4 degrees, with a screen resolution of 1024 × 768 (after scaling to 350 × 350 pixels). Words and pseudowords were presented in lower case Helvetica. Their visual angle ranged from 1.47 to 4.41 degrees with the majority of words (with 5 letters) extending 1.84 to 2.2 degrees. Auditory stimuli were presented using headphones designed to filter in-scanner noise (MR Confon, Magdeburg, Germany). Volume was adjusted for each participant to maximise audibility during a practice task of single word repetition, in which it was confirmed that participants could hear the stimuli over the scanner noise.

#### 2.3.3 Behavioural response acquisition

Spoken responses were captured using a noise-cancelling MRI compatible microphone (FOMRI III^TM^ Optoacoustics, Or-Yehuda, Israel). They were transcribed at the time of imaging and scored for accuracy off-line by listening to the audio files. Each response was categorised as correct (when the response matched the target) or incorrect (when the response did not match the target and/or was delayed).

Reaction times (RTs) for spoken responses were obtained from the audio files using an adaptive moving window filter that was tailored to each participant. The optimal window length (i.e. the width which maximally smoothed the audio stream) was based on a portion of the audio file collected during the resting baseline between blocks of stimuli. After smoothing the whole time series, we defined the onset of speech as a rise in the absolute amplitude of the smoothed audio stream beyond 3 standard deviations from the mean. We analysed the average RT per condition per participant, for correct trials only. Due to technical issues, RTs were missing for 7% of the data points (23/328) from 41 participants performing 8 different tasks (2 from POI, 11 from PC; and 7 from NC, with 3 participants missing 2 data points).

### 2.4 Acquisition of MRI data

MRI data were acquired on a 3T PRISMA Siemens scanner. Anatomical high resolution T1-weighted structural images were acquired using a 3D magnetization-prepared rapid acquisition gradient-echo (MPRAGE) sequence with a voxel size of 1 × 1 × 1 mm (TR / echo time (TE) / Inversion Time = 2530 / 3.34 / 1100 ms, flip angle = 7 degrees, matrix size = 256 × 256, 176 slices). Functional images were acquired using a gradient EPI sequence with 3 × 3 mm in-plane resolution (TR / TE = 3080 / 30 ms, flip angle = 90 degrees, field of view = 192 mm, matrix size = 64 × 64, slice thickness = 2.5 mm, inter-slice gap = 0.5 mm, 44 axial slices). 66 image volumes per session were acquired, including 5 dummy scans to allow for magnetisation to reach equilibrium. The TR was chosen to maximize whole brain coverage and to ensure that slice acquisition onset was offset with stimulus onset, which allowed for distributed sampling of slice acquisition across the study (Veltman et al., 2002). As inferior cerebellar regions might have been under-represented in previous fMRI studies, we ensured that data were acquired from the whole of the cerebellum.

### 2.5 Processing of structural MRI data

The T1-weighted anatomical whole-brain volume of each participant was analysed with an automated lesion identification toolbox (Seghier et al., 2008). The toolbox is a modification of the unified segmentation–normalisation routine implemented in SPM8, which has been shown to be more accurate and robust compared to other methods, when dealing with lesioned brains (Crinion et al., 2007). The toolbox converts a scanner-sensitive raw image into a quantitative assessment of structural abnormality by first segmenting the whole brain into four tissue classes: grey matter, white matter, and cerebrospinal fluid, as used in the standardised routine, and an additional atypical tissue class. This fourth class represents outlier voxels within grey and white matter that are far from the normal range of the voxel values in controls. The tissue affected by a lesion will therefore be identified as an outlier and classified as atypical. Modelling the existence of the lesion explicitly as an extra class during iterative brain segmentation helps to avoid misclassification of damaged voxels. The output is a binary image that delineates the lesion(s) and here was used to estimate lesion volume, visualise the lesions of the patient controls, and calculate the degree of damage to regions of interest. All automatically generated lesion images were inspected by eye, and compared to the lesion description reported by a neurologist. The cluster size threshold for lesion identification was initially set at the default (100 contiguous voxels) for all lesions. However, this threshold failed to identify some small lesions, so we reduced the cluster extent threshold to 20 contiguous voxels and visually inspected the results. One author (LMS) manually deleted artefacts generated at this lower threshold. Artefacts were defined as regions where the automated software identified a lesion which was not described in the neurologist report.

### 2.6 Processing of functional MRI data

Data pre-processing was performed in the Statistical Parametric Mapping software (SPM12; Wellcome Centre for Human Neuroimaging, London, UK; https://www.fil.ion.ucl.ac.uk/spm/), running in MATLAB environment (2018a Mathworks, Sherbon, MA, USA). Functional volumes were spatially realigned to the first EPI volume and unwarped to compensate for non-linear distortions caused by head movement or magnetic field inhomogeneity. We used the unwarping procedure, in which the interaction between head movement and any inhomogeneity in the T2* signal is modelled. To spatially normalize all realigned EPI scans to the MNI standard space, we co-registered the mean EPI image to the anatomical T1 image, spatially normalized the anatomical image using the new unified segmentation-normalization routine, and applied the deformation field parameters to the EPI images. The original resolution of the images was maintained during normalization. After the normalization procedure, the functional images were spatially smoothed with a 6 mm full-width half-maximum (FWHM) isotropic Gaussian kernel to compensate for residual anatomical variability and to permit application of Gaussian random-field theory for statistical inference (Friston et al., 1995). Each pre-processed functional volume was individually inspected for oddities before statistical analyses.

For the first level analysis of each participant, data from each task were entered into a subject-specific fixed-effect analysis using the general linear model (GLM; Friston et al., 1995). Stimulus functions were convolved with a canonical hemodynamic response function. To exclude low-frequency confounds, the data were high-pass filtered using a set of discrete cosine basis functions with a cut-off period of 128 s. We maximised brain coverage by including all voxels whose mean value is at least 20% of the global signal. All stimulus onset times were modelled as single events. Correct, incorrect, and no responses were modelled separately, and only the correct responses were submitted to the second level analysis. Contrasts submitted to the second level analysis therefore characterised the activation difference between correct trials and the resting baseline.

### 2.7 Normal speech production regions and selection of patients of interest

To determine whether patients with cerebellar lesions had damage to the normal speech production system (our ‘patients of interest’ group), we first identified speech production regions in the cerebellum where neurologically intact controls showed activation during all 8 speech production tasks (second level analysis with one group of all neurologically intact controls, 8 contrasts (each Task − Rest), and no covariates). The voxel-level statistical threshold was set at p < 0.05, after Family Wise Error (FWE) correction for multiple comparisons across the whole brain, with no minimum cluster size. Four cerebellar regions were significantly activated across all 8 speech production tasks, in the neurologically intact control group: bilateral cerebellar lobules V/VI (x = −21, y = −58, z = −22; x  = 21, y = −58, z = −22) and bilateral inferior portion of the posterior cerebellum (Crus II / VIIb / VIIIa; x  = −21, y = −64, z = −43; x  = 18, y = −73, z = −43), see Fig. 1 and Supplementary Table 2.

Patients of interest were therefore identified as those for whom the automatically generated binary lesion image overlapped, to any extent, with the normal cerebellar speech production regions (average overlap: 7 ± 12%, range: 1–34%). Patients who did not have damage overlapping with the normal cerebellar speech production regions were assigned to the patient control group. For PS0472 (whose lesion was not detected by the lesion identification software), we verified by eye that the lesion was in the left ventral cerebellar Crus II, and did not overlap with any of the normal cerebellar speech production regions. For speech production regions outside the cerebellum see Supplementary Material.

### 2.8 Speech production activation after damage to cerebellar speech production regions

Using a factorial design with 3 independent groups of participants (POI, PC and NC), 8 tasks, no covariates, and a voxel-level statistical threshold of p < 0.05 FWE-corrected for multiple comparisons across the whole brain, we identified (i) enhanced activation for patients of interest (POI) where activation was significantly higher across all 8 speech production conditions in POI, compared to NC; and, (ii) reduced activation in POI using the reverse contrast. To ensure that group differences were driven by activation in the group of interest rather than deactivation in the comparison group, we only report greater activation in the group of interest, where there was also activation across all 8 speech production conditions compared to rest in the group of interest (at p < 0.001 uncorrected). This was achieved using the inclusive masking option in SPM. For example, the contrast [POI > NC] was inclusively masked with the contrast [All 8 Tasks in POI minus Rest], at a voxel-level statistical threshold of p < 0.001 uncorrected, to ensure that we only report regions where POI had increased activation during the speech production tasks. Similarly, the contrast [NC > POI] was masked with the contrast [All 8 Tasks in NC minus Rest], at a voxel-level statistical threshold of p < 0.001 uncorrected.

When a significant group difference (POI versus NC) was detected at a whole brain FWE-corrected level, we further report group difference comparing POI and PC. The search volume included the entire cluster where activation was significantly different between POI and NC (FWE-corrected), without applying small volume correction. Differences between POI and PC are reported at a statistical threshold of p < 0.001 uncorrected to investigate, at a less conservative threshold, whether abnormal activation was lesion site dependent.

### 2.9 Are effects of interest dependent on the behavioural task?

To investigate whether activation in the normal cerebellar speech production regions is influenced by task parameters, we first tested whether cerebellar activation in neurologically intact controls interacted with the task related factors. We report on main effects of task from a full factorial analysis with one group (all neurologically intact controls), 3 factors (visual versus auditory stimuli; verbal versus nonverbal stimuli; semantic versus non-semantic content), and no covariates. We also tested for the effect of Lexicality (words or pseudowords). Effects are reported at a statistical threshold of p < 0.05 after voxel-level FWE-correction for multiple comparisons across the whole brain.

We then tested whether regions demonstrating group effects (areas of significantly higher/lower activation in POI compared with NC) show an interaction between Group (NC and POI) and the task-related factors. The search volume included the entire cluster where activation was significantly different between POI and NC (FWE-corrected), without applying small volume correction. We report these results at a voxel-level threshold of p < 0.001 uncorrected, to investigate, at a less conservative threshold, whether abnormal activation was task dependent.

### 2.10 Inter-patient variability in activation among patients

The goal here was to examine whether differences in activation between patients of interest and patient controls could be partially explained by patient characteristics, rather than the lesion sites used to define these two patient groups (i.e. POI versus PC). We examined the variability in each of the regions where speech production activation (across all 8 tasks) was significantly higher/lower for patients of interest compared to neurologically intact controls. Using a 3 mm radius region of interest centred on the peak coordinates for these group differences, we extracted the principal eigenvariate across voxels for each of the 8 tasks minus rest. We averaged the activation across all tasks and studied the association between activation and the following variables:

(i) Lesion location: we assigned the patient controls to sub-groups based on whether their lesions affected the dominant hemisphere (left cerebrum and/or right cerebellum; n = 10); the non-dominant hemisphere (right cerebrum and/or left cerebellum; n = 8); or both (the bilateral cerebrum group; n = 2, was not included in further analysis due to insufficient data). See Supplementary Table 1 for grouping. Activations were compared using one-way MANOVA (3 groups, 2 sites of abnormal activation) followed by post-hoc independent sample t-tests.
(ii) Damage to the normal speech production regions: Patient controls with damage to the normal speech production regions were identified as those for whom the automatically generated binary lesion image overlapped, to any extent, with the whole-brain normal speech production regions (i.e. areas of significant activation associated with all 8 speech production tasks in neurologically intact controls using a voxel-level statistical threshold of p < 0.05 FWE-corrected across the whole brain, as defined above). This resulted in two groups: patient controls with damage to cerebral cortical speech production regions (n = 10), and patient controls with no damage to cerebral cortical speech production regions (n = 10), see Supplementary Table 1 for grouping. These two groups were compared to the patients of interest with damage to cerebellar speech production regions (n = 7). Activations were compared using one-way MANOVA (3 groups, 2 sites of abnormal activation) followed by post-hoc independent sample t-tests.
(iii) Time between stroke and test, age at test, lesion volume and in-scanner performance (accuracy level or RT). Here we used two-tailed Pearson’s correlation with Benjamini-Hochberg correction for multiple comparisons using the false discovery rate. Variability across all patients (n = 27), and in each patient group, was studied separately. All data were analysed in IBM SPSS Statistics for Windows, Version 26.0 (IBM Corp., Armonk, New York, USA).

## 3 Results

### 3.1 Articulation abilities

At 1 month post-stroke, six of the seven patients of interest reported having speech impairments affecting articulation (see Table 3). The exception was PS1575, who reported only having word finding difficulties acutely. We report effects including all 7 patients and confirmed that the results did not change when PS1575 was excluded. Information about speech abilities of patient controls at 1 month post-stroke is provided in Supplementary Table 3.

**Table 3:** Speech abilities and therapy post-stroke for patients of interest.

Patient ID	Acute		Chronic		SLT
	Self-rating	Notes	Self-rating	Notes	
PS2068	Short sentences	Problems sequencing words, sounds like tongue is lolling when speaking	Short to normal sentences	Slurred speech	–
PS2464	Short sentences	Initially slurred speech, better after SLT, but slurs when tired/fatigued	Short to normal sentences	Advanced speech but not back to normal	Once a week for 2 months, tongue exercises, repetition
PS1327	Normal sentences	Difficulties with speech, slurred speech	Normal sentences	–	< 20 h
PS2504	Short sentences	Swallowing difficulties, hoarse voice, struggling to vocalise. “My voice sounds different to me, I have a feeling of something stuck in my throat.”	Short to normal sentences	–	<20 h, word finding, tapping to the rhythm of words
PS0573	Using gestures but not speaking at all	Dysarthria, dysphagia	Short sentences	–	50 – 100 h
PS1575	Short to normal sentences	–	Short to normal sentences	Sometimes says the wrong word	–
PS0452	Short sentences	Muddled speech and reduced intonation	Short to normal sentences	Not back to normal, can only produce short sentences	–

“-“ = no notes describing speech abilities / SLT; Acute = 1 month post-stroke; Chronic = at time of testing; SLT = Speech and Language Therapy. Self-rating: patients retrospectively evaluated their speech abilities, indicating on a scale whether they were unable to attempt speech, speak or use gestures; or able to use gestures but not speak; use only 1 or 2 single words; use a few single words; speak in short sentences; or speak normally.

At the time of CAT testing (≥3 months post-stroke), analysis of the patients’ speech during the Spoken Picture Description task using the Apraxia Battery for Adults inventory showed that none of the patients had a score indicative of apraxia of speech (i.e. score ≥5). In fact, dysarthric or apraxic behaviours were rare in our dataset: 1 patient of interest and 4 patient controls had a score of 1, indicating one occurrence of dysarthric/apraxic behaviour, while the others had a score of 0 (see Supplementary Table 4, ‘Articulation errors’ in Spoken Picture Description).

On the single word Naming and Repetition tasks, three of the patients of interest exhibited a small number of articulation errors (PS2464 omitted 'n' from a final consonant cluster, repeating ‘president’ as 'presidud', and naming ‘elephant’ as ‘elephut’; PS2068 named ‘camera’ and ‘caravan’ as 'camwa’ and ‘cawavan'; and PS2504 repeated ‘president’ as 'presid', followed by a self-correction), while one patient control exhibited mild dysarthric behaviours (PS1627 named ‘pyramid’ and ‘frog’ as 'pywamid’ and, ‘fwog'), see Supplementary Table 4, ‘Articulation errors’ in Naming and Repetition.

Speech production during the fMRI scan (>6 months after stroke) involved errors of all types (articulation, semantic, phonological, etc) across both patient groups, with 3 (out of 7) patients of interest and 10 (out of 20) patient controls exhibiting at least one articulatory error. As with the CAT tasks, errors during the fMRI tasks were rare, with patients of interest producing articulation errors, on average, on 1.8% (±2.9) of trials, and patient controls producing articulation errors on 1.3% (±1.8) of trials (see Supplementary Table 4, ‘Articulation errors’ in fMRI).

A summary of all patients’ articulation and other errors on the CAT and fMRI tasks is available in Supplementary Table 4.

### 3.2 In-scanner performance

The patients of interest were able to accurately produce speech in the scanner, with >94% accuracy, on average, for all but one of the 8 speech production tasks. The exception was naming sounds because 3 patients of interest had relatively low accuracy (62.5%, 70% and 72.5%). However, some of the patient and neurologically intact controls also had difficulty with the sound naming condition (see Supplementary Table 5A), and consequently, there were no significant differences in accuracy between patients of interest and either control groups (PC or NC, independent sample t-tests, p > 0.05) for any of the speech production tasks. There were also no significant differences in response times for the patients of interest and the neurologically intact controls for any of the tasks (independent sample t-tests, p > 0.05), but the patient controls were significantly slower than the patients of interest in repeating words and pseudowords (t_(24)_ = 2.95, p = 0.007; t_(21)_ = 3.51, p = 0.002, respectively; see Supplementary Table 5B for further details on group RTs).

### 3.3 Speech production activation after damage to cerebellar speech production regions

**Fig. 3:**
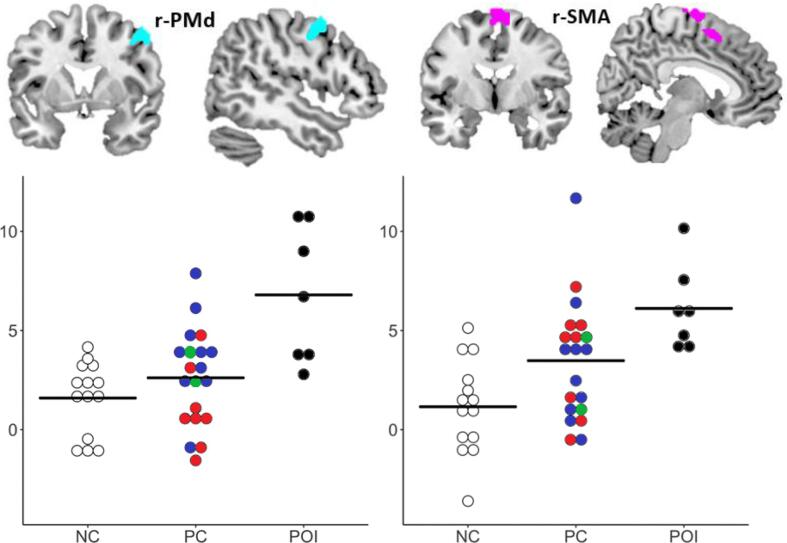
Inter-subject variability in regions showing enhanced activation in the patients of interest. Top: Anatomical location in MNI space and activation in r-PMd (cyan, left) and r-SMA (purple, right), where activation was significantly higher in patients of interest (POI; black) compared to neurologically intact controls (NC; white), at voxel-level threshold of p < 0.05 FWE-corrected across the whole brain. Clusters are displayed at voxel-level threshold of p < 0.001 uncorrected. At this lower threshold the r-SMA activation extends into the left SMA. Bottom: In the patient controls group (PC) individuals are coded according to lesion location (see Supplementary Table 1): Blue = left cerebrum and/or right cerebellum (n = 10), Red = right cerebrum and/or left cerebellum (n = 8), Green = bilateral cerebrum (n = 2). Horizontal reference lines represent average activation for each group. Y axis represents the principal eigenvariate in the corresponding region.

Compared to the neurologically intact controls, patients with damage to the cerebellar speech production regions (POI) showed enhanced activation (across all 8 conditions, p < 0.05 FWE-corrected) in two regions: right dorsal premotor cortex (r-PMd) and right supplementary motor area (r-SMA) (see Table 4 and Fig. 3).

**Table 4:** Enhanced activation in the patient of interest group compared with neurologically intact controls.

Region	Hemisphere	MNI Coordinate			Z-score	Cluster size p < 0.001 unc.
		x	y	z		
r-PMd	Right	48	5	50	5.50	72
r-SMA	Right	6	−4	74	4.71	166

PMd = dorsal premotor cortex; SMA = supplementary motor area.

As both regions were not lesioned in any of the patient controls, we did not exclude any patient controls from further analysis. In r-PMd, activation was also higher in patients of interest than patient controls (Z = 4.51, p < 0.001 uncorrected). In r-SMA, the difference between patients of interest and the patient control group was not significant (p > 0.001 uncorrected). The group difference for [POI > PC] was smaller than that for [POI > NC] because some of the control patients also had enhanced activation, relative to NC, in r-PMd and r-SMA (see Fig. 3). The lesion sites and fMRI results for these patients are described in the Supplementary Material (section ‘Enhanced r-PMd and r-SMA activation in patient controls’).

No clusters were significantly less activated for patients of interest compared with neurologically intact controls, even at p < 0.001 uncorrected. This is not surprising given the variation in lesion site across the patients of interest. Below, we investigate behavioural and task effects to further understand the differences in activation between the patients of interest and neurologically intact controls.

### 3.4 Is enhanced activation in r-PMd and r-SMA dependent on the task?

**Fig. 4:**
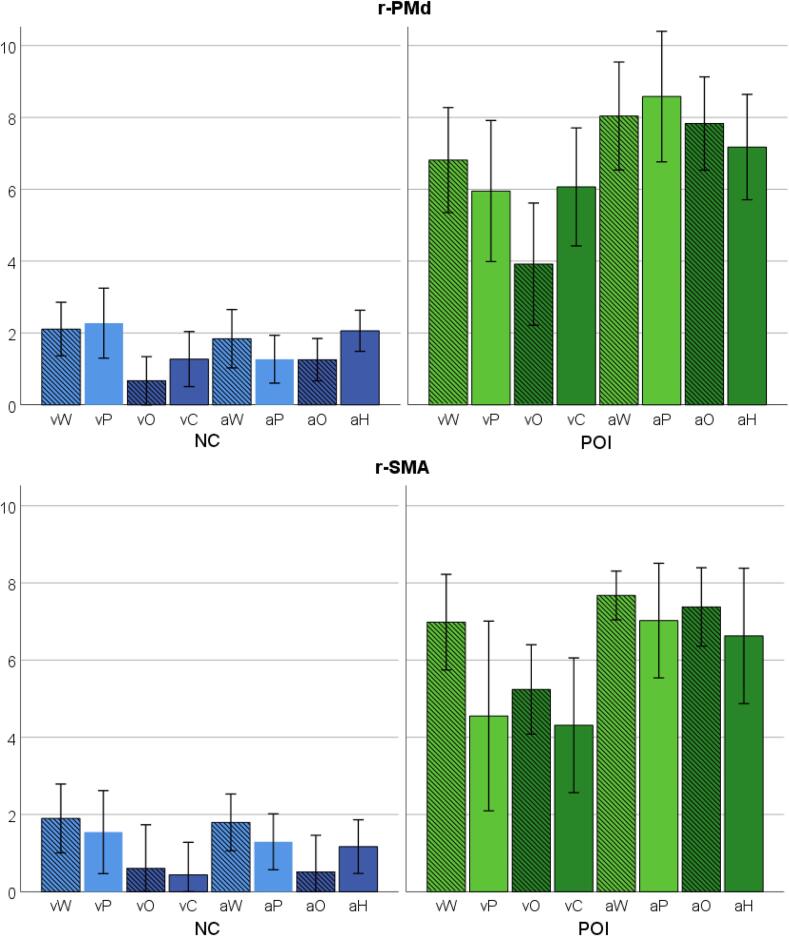
Task dependent responses in r-PMd and r-SMA. Activation during tasks differing in Modality (Visual = v / Auditory = a), verbal content (Verbal = light colour / Non-verbal = dark colour), and Semantic content (Semantic = stripes / Non-semantic = no stripes), in the right dorsal premotor cortex (r-PMd, Top) and right supplementary motor area (r-SMA, bottom). Y axis represents the principal eigenvariate extracted from 3 mm radius spheres centred on the peak coordinates reported in Table 4, for neurologically intact controls (NC, Blue) and patients of interest (POI, Green). Error bars represent ± 1 standard error. From left to right: word reading (vW); pseudoword reading (vP); object naming (vO); colour naming (vC); word repetition (aW); pseudoword repetition (aP); object naming (aO); naming gender of humming voice (aH).

In the neurologically intact controls, activation in the cerebellum showed no significant effects of (i) stimulus modality (auditory versus visual), (ii) verbal content (words and pseudowords versus objects and baselines), (iii) semantic content (words and objects versus pseudowords and baselines), or (iv) lexicality (words versus pseudowords); (p > 0.05 FWE-corrected for all). Accordingly, there was also no significant interaction between group (POI and NC) and these task factors in r-PMd or r-SMA (p > 0.001 uncorrected for all). See Fig. 4.

### 3.5 Inter-patient variability in activation

The plots in Fig. 3 show inter-patient variability within and across groups. Below we explore variables which might influence this inter-patient variability.

(i) Lesion location: a one-way MANOVA of activation in r-PMd and r-SMA among the three groups of patients (i.e. patients of interest with damage to cerebellar speech production regions, patient controls with damage to left cerebrum and/or right cerebellum, and patient controls with damage to right cerebrum and/or left cerebellum, see Fig. 3 for details), revealed no significant effect of region (F_(1,22)_ = 1.51, p = 0.232), but a significant main effect of group (F_(2,22)_ = 5.63, p = 0.011), and a significant interaction between region and group (F_(2,22)_ = 13.14, p = 0.045). Post-hoc tests showed that POI had higher activation in the r-PMd compared to patients with: (i) right cerebrum and/or left cerebellar lesions (n = 8, red in Fig. 3, t_(13)_ = 4.04, p = 0.001), and (ii) left cerebrum and/or right cerebellar lesions (n = 10, blue in Fig. 3, t_(15)_ = 2.19, p = 0.045). Patient controls with left cerebrum and/or right cerebellum damage had higher r-PMd activation compared to patient controls with right cerebrum and/or left cerebellar damage (t_(16)_ = 2.62, p = 0.019). Higher activation for the POI group compared with the two patient controls sub-groups cannot be explained by lesion size, which was largest in the patient controls with right cerebral lesions.

There was no significant difference in activation between any patient groups in the r-SMA (p > 0.05 for all comparisons). We did not compare the patients of interest to the sub-group of patient controls with bilateral cerebral damage, as there were only two such patients (green in Fig. 3).

(ii) Damage to normal speech production regions: a one-way MANOVA of activation in r-PMd and r-SMA among the three groups of patients (i.e. patients of interest with damage to cerebellar speech production regions, patient controls with damage to cerebral speech production regions and patient controls with no damage to any of the speech production regions) revealed a significant main effect of group (F_(2,24)_ = 5.97, p = 0.008), but no significant effect of region (F_(1,24)_ = 0.72, p = 0.405) or interaction between region and group (F_(2,24)_ = 1.56, p = 0.230). Post-hoc tests showed that patients of interest had significantly higher activation compared to patients with damage to cerebral speech production regions (t_(15)_ = 3.53, p = 0.003), and compared to patients with no damage to speech production regions (t_(15)_ = 2.29, p = 0.037). Patients controls with and without damage to cerebral speech production regions did not differ from each other (t_(18)_ = 1.08, p = 0.295).

(iii) Time between stroke and testing, age at testing, lesion volume, and in-scanner performance (accuracy level or RT): none of these variables significantly correlated with activation in either r-PMd or r-SMA (Pearson’s r, non-significant p for all), when all patients were pooled together, or within each patient group. The lack of significant correlation within each patient group may reflect the small sample size of these groups. Further details of the (non-significant) relationships between activation and the above variables, within the patient of interest group, are illustrated in Supplementary Fig. 2.

## 4 Discussion

In this study we investigated brain activation in stroke survivors with unimpaired speech measured by a standardised clinical test battery, years after sustaining damage to cerebellar speech production regions. We expected to observe enhanced activation in one or more undamaged cerebral and/or cerebellar regions during accurate speech production and indeed found abnormally high activation in two cerebral regions: right PMd and right SMA. Six of the seven patients of interest had a lesion affecting the right cerebellum, and as hypothesised, enhanced activation was found in undamaged parts of the ipsilesional cerebral cortex (i.e. r-PMd and r-SMA), as reported by Kinomoto et al. (2003). The right PMd and right SMA are typically considered to belong to the canonical speech production system and share structural connections through association fibres (Hartwigsen et al., 2013). Below we discuss how activation in the cerebellar speech production regions, r-PMd, and r-SMA depend on patient group; and how our findings provide insights into the potential mechanisms that support accurate speech production after damage to cerebellar speech production regions.

### 4.1 The regions of the cerebellum involved in speech production

Our cerebellar speech production regions of interest were identified, in neurologically intact controls, in bilateral lobules V/VI, and a region spanning bilateral Crus II, lobule VIIb and lobule VIIIa. These results are in line with numerous previous studies of articulation (Bohland and Guenther, 2006, Correia et al., 2020, Peeva et al., 2010, SHUSTER and LEMIEUX, 2005), and a recent somatotopic mapping study of the cerebellum (Boillat et al., 2020) which showed that the activation we documented in the cerebellum is centred on the face representation. Our findings are also in line with Guell et al.’s (2018b) description of two motor representations in the cerebellum, one in bilateral lobules I-VI and the second in bilateral lobule VIII.

Each of our patients of interest had a focal lesion to one or more of these cerebellar speech production regions (Table 1 and Fig. 1). Six of the seven patients of interest had speech impairments acutely, according to medical notes and their retrospective speech self-rating, but none were significantly impaired when tested years post stroke, according to standardised tests of articulation ability. The occurrence of impairments following lesions to various sites in the cerebellum, and the subsequent recovery, are both in line with previous accounts of transient speech production impairments following varied cerebellar stroke lesions (Ackermann et al., 1992, Schweizer et al., 2010, Zettin et al., 1997). It has also been suggested that recovery from cerebellar lesions may be incomplete, with residual deficits that are too subtle to be detected by standardised assessment batteries (Fabbro et al., 2000, Mariën and Beaton, 2014). Irrespective of whether or not this is the case, we have exclusively reported brain activation related to successful speech production because only accurate overt speech responses were considered in our fMRI analyses.

One of the patients of interest with damage to the normal cerebellar speech production regions (PS1575) reported having no speech impairments either acutely or chronically. This can be a result of individual variability in functional anatomy. If this individual was not using the region of the cerebellum affected by the stroke for speech production premorbidly, then indeed no impairment would be expected following stroke. A related issue is that PS1575 is the only patient of interest who is left-handed. While the normal speech production activation in the cerebellum was found to be bilateral, it was not symmetric, especially in the posterior lobules where it was right-lateralised (see Fig. 1). Thus, if the patient had premorbid atypical language lateralisation associated with left-handedness, then the lesion might only minimally affect the speech production system, or even not at all. Still, we note that all analyses reported here remained the same when excluding this patient.

Finally, while none of the patient controls had lesions affecting the normal speech production regions in the cerebellum, three (PS1627, PS0369 and PS1343) had cerebellar damage in close proximity (affecting Crus II, lobule VIIb and/or lobule VIIIa), and in fact, their lesions partly overlapped with that of one of the patients of interest (PS1575). Interestingly, by examining the activation profiles in r-PMd and r-SMA of these three patient controls, it was observed that the level of activation for PS1343 and PS1627 was below the lowest value obtained in the patients of interest group, and that for PS0369 was well below the average of the patients of interest. While this observation is anecdotal, it provides no evidence that the cerebellar regions affected in these three patient controls were associated with higher than normal activation in r-PMd or r-SMA. This gives further support to our suggestion that enhanced activation in the two cerebral cortical motor regions is likely to be the consequence of damage to normal cerebellar speech production regions.

### 4.2 The role of the dorsal premotor cortex during speech production

Our finding that accurate speech in patients with cerebellar damage co-occurs with greater activation of right PMd is the first to suggest that this region could compensate for reduced input from the cerebellum during speech production. This hypothesis is consistent with prior evidence, from a TMS study, that higher PMd activation has a beneficial role on (hand) movement control (Johansen-Berg et al., 2002). In addition, we have illustrated high inter-patient variability in right PMd activation within and between groups (Fig. 3) even though none of our patients had damage to the PMd region. Our findings are therefore consistent with the observations of a prior study showing that activation during speech production varies according to lesion site (Skipper-Kallal et al., 2017).

In terms of how r-PMd activation could compensate for cerebellar damage, we note that several prior studies have reported bilateral PMd activation for both speech and hand movements (Price, 2012), with activation increasing during attention demanding or complex motor coordination tasks; for example, when speaking and finger tapping occur at the same time compared to either speaking or finger tapping alone (Meister et al., 2009). Enhanced r-PMd activation in our cerebellar patients of interest may therefore reflect a response to increased demands on the motor control system.

Future longitudinal studies are now required, with larger patient cohorts to (i) understand the factors that determine when r-PMd activation changes after stroke; (ii) characterize how exactly r-PMd contributes to accurate speech production after cerebellar damage; and, (iii) identify alternative compensatory systems for those patients who did not show enhanced activation in r-PMd.

### 4.3 The role of the supplementary motor area during speech production

Activation in r-SMA was also higher in our patients of interest than the neurologically intact controls. However, enhanced r-SMA activation was not specific to these patients because it did not differ from that in patient controls. Other studies of speech processing after stroke have also reported enhanced SMA activation during overt speech production in patients with left inferior frontal lesions (Rosen et al., 2000) and during the recovery of speech comprehension in patients with a range of different left hemisphere lesions (Stockert et al., 2020). Likewise, Obayashi (2020) reported that SMA activation was higher in patients with thalamic lesions who had better verbal fluency. Additionally, enhanced SMA activation after stroke is not limited to speech tasks. For example, Kenzie et al. (2019) found that bilateral SMA (and premotor) activation increased with performance on a proprioceptive–dependent motor task.

Together these studies suggest that SMA might be able to compensate when there is damage to the motor execution network. Anatomically, this can be supported by the rich connections that SMA has with multiple parts of the motor system, such as the primary motor cortex (Kim et al., 2010) and basal ganglia (Liu et al., 2020). Functionally, the SMA has been shown to receive preparatory signals on the order of information from pre-SMA and to send driving signals to the motor regions that execute motor commands (Bohland et al., 2010).

In terms of how SMA activation could compensate for cerebellar damage, prior studies suggest that the SMA is involved in higher motor function (Picard and Strick, 1996), in particular movement preparation (Nachev et al., 2008), including the motor programming of articulation (Peeva et al., 2010), and non-speech oral movements (Basilakos et al., 2018), even when cognitive demand is low (Cummine et al., 2017). It is therefore possible that enhanced SMA activation after damage to the motor system (such as in our cerebellar patients of interest), reflects increased demands on motor programming.

### 4.4 Limitations and future directions

As in other studies of patients with focal cerebellar strokes (Fabbro et al., 2000, Marien et al., 2000), our group sample sizes were relatively small, even though the total number of patients (27) was relatively high. The difficulty recruiting patients with cerebellar lesions for research has been explained by the rarity of cerebellar strokes (∼2% of total strokes according to Tohgi et al., 1993), as well as the disproportionally high mortality rate associated with such stroke on the one hand, and more subtle manifestation of clinical symptoms among stroke survivors on the other hand (Macdonell et al., 1987, Nickel et al., 2018, Tohgi et al., 1993). We faced an additional challenge insofar as our patients of interest are only those who had focal damage affecting parts of the cerebellum that are involved in speech production.

A second point to note is that, although we focused on patients with damage to speech production regions, there was variability in the precise location of the lesions in our patients of interest. This variability might explain why we did not observe compensatory activation within the cerebellum as observed for a single bilingual patient with left cerebellar damage, when naming in the non-native language (Mariën et al., 2017), and in patients who recovered their hand motor function following cerebellar stroke (Kinomoto et al., 2003). Future studies are therefore required to compare the effect of damage to different cerebellar regions on both speech production performance and brain activation.

Thirdly, the initial speech impairments, in the acute stage after stroke, could only be described based on medical notes and retrospective rating by the patients themselves. Future studies could determine the relationship between speech impairment level at the acute stage, and compensatory systems seen at the chronic stage, by acquiring more precise behavioural data at the acute stage and following the patients longitudinally.

Lastly, more advanced methodological approaches could be used in future studies to acquire more fine grained data with higher spatial and temporal resolution.

### 4.5 Conclusions

To our knowledge, this is the first study to demonstrate enhanced activation in cerebral motor regions (right PMd and right SMA) during accurate speech production in patients with cerebellar damage to the speech production network. Both right PMd and right SMA are known to be part of the speech system and their increased activation may plausibly play a role in functionally compensating for damage to cerebellar and non-cerebellar speech production regions. Our findings therefore motivate future studies to test whether non-invasive neurostimulation to our r-PMd and r-SMA regions of interest impairs speech production in chronic stroke patients with damage to cerebellar speech production regions more than in neurologically intact controls.

## Funding

This work was supported by the Wellcome Trust (203147/Z/16/Z and 205103/Z/16/Z to C.J.P.); the Medical Research Council (MR/M023672/1 to C.J.P); and the Stroke Association (TSA 2014/02 to C.J.P. and D.W.G., TSA PDF 2017/02 to T.M.H.H.).

## CRediT authorship contribution statement

**Sharon Geva:** Conceptualization, Methodology, Formal analysis, Writing – original draft, Writing – review & editing, Visualization, Supervision. **Letitia M. Schneider:** Methodology, Formal analysis, Investigation, Data curation, Writing – original draft, Writing – review & editing, Visualization. **Sophie Roberts:** Formal analysis, Writing – review & editing. **Shamima Khan:** Investigation, Resources, Data curation, Project administration. **Andrea Gajardo-Vidal:** Investigation, Formal analysis, Data curation, Writing – review & editing. **Diego L. Lorca-Puls:** Investigation, Formal analysis, Data curation, Writing – review & editing. **PLORAS team:** Investigation, Resources, Project administration. **Thomas M.H. Hope:** Software, Writing – review & editing. **David W. Green:** Methodology, Writing – review & editing, Supervision, Funding acquisition. **Cathy J. Price:** Conceptualization, Methodology, Writing – original draft, Writing – review & editing, Supervision, Project administration, Funding acquisition.

## Declaration of Competing Interest

The authors declare that they have no known competing financial interests or personal relationships that could have appeared to influence the work reported in this paper.
