# Supplementary material for: Right cerebral motor areas that support accurate speech production following damage to cerebellar speech areas
Source: Neuroimage Clin. 2021 Sep 20;32:102820. doi: 10.1016/j.nicl.2021.102820 (PMC8517928; doi:10.1016/j.nicl.2021.102820)
Supplement: Supplementary data 1 [file mmc1.docx]

**Supplementary Material**

# Participants

**Table 1.** Demographics and testing details of patient controls.

| **Patient ID** | **Lesion location** | **SP*** | **Age at stroke** | **Gender** | **Lesion vol. (cm^3^)** | **Hand** | **Stroke to CAT (months)** | **Stroke to fMRI (months)** |
| --- | --- | --- | --- | --- | --- | --- | --- | --- |
| **L Cerebrum / R Cerebellum** | | | | | | | | |
| PS0451 | L white and grey matter deep to the insula | N | 49.3 | M | 8.2 | R | 28 | 124 |
| PS2879 | L posterior putamen | N | 38.0 | F | 0.7 | R | 4 | 24 |
| PS2884 | L putamen and caudate; posterior thalamus (pulvinar) | N | 69.1 | M | 1.2 | R | 5 | 24 |
| PS3021 | L sub-insular, putamen | N | 52.5 | M | 13.7 | R | 20 | 20 |
| PS3476 | L sub-insula, putamen | N | 57.3 | M | 11.7 | R | 60 | 60 |
| PS3488 | L insula and putamen | N | 49.1 | M | 9.9 | L | 17 | 16 |
| PS2386 | L post-central gyrus, R cerebellar Crus I lacune | Y | 59.8 | M | 2.0 | R | 50 | 50 |
| PS1627 | L medial occipital; R cerebellar Crus II, VIIIb | N | 44.5 | M | 1.9 | R | 12 | 25 |
| PS0369 | R cerebellar Crus II, VIIb, VIIIa/b, IX, olivary nucleus | N | 31.1 | F | 7.8 | R | 110 | 225 |
| PS1343 | R cerebellar Crus II, VIIb, VIIIa/b, IX | N | 44.6 | M | 3.3 | R | 71 | 99 |
| **R Cerebrum / L Cerebellum** | | | | | | | | |
| PS2003 | R MCA | Y | 35.4 | F | 117.0 | R | 58 | 98 |
| PS2797 | R MCA/PCA | Y | 47.6 | M | 110.1 | R | 18 | 26 |
| PS2872 | R MCA | Y | 46.2 | M | 125.4 | R | 22 | 33 |
| PS2981 | R MCA | Y | 50.3 | F | 151.6 | R | 37 | 37 |
| PS3189 | R MCA | Y | 57.6 | F | 84.3 | Ambi | 3.2 | 38 |
| PS3235 | R posterior MCA | Y | 60.0 | M | 51.6 | R | 104 | 104 |
| PS3608 | R temporo-parietal | Y | 62.1 | M | 21.0 | L | 5 | 10 |
| PS0472 | Left cerebellum Crus II | N | 39.0 | F | 0 | R | 125 | 224 |
| **Bilateral cerebrum** | |  |  |  |  |  |  |  |
| PS0673 | L ventral and posterior IFG and posterior insula; R medial occipital lobe | Y | 54.3 | M | 62.8 | R | 148 | 219 |
| PS3315 | L dorsal MCA: frontal and parietal lobes, head of caudate; R premotor cortex | Y | 39.2 | M | 128.4 | R | 58 | 57 |

* Damage to speech production (SP) regions (see section 2.10 – ii): Y = yes, N = no.

Lesion: IFG = inferior frontal gyrus, L = left, MCA = middle cerebral artery, PCA = posterior cerebral artery, R = right; Hand = dominant hand: Left (L), Right (R) or ambidextrous (Ambi); Gender = Male (M) or Female (F).

# Speech production regions outside the cerebellum

Other parts of the brain activated by neurologically intact controls during single word production (p < 0.05 after FWE correction for multiple comparisons across the whole brain) were located bilaterally in the pre/post-central gyrus, posterior and middle portions of the superior temporal gyrus (STG), supplementary and pre-supplementary motor area (SMA / pre-SMA), right thalamus, left anterior STG, and bilateral medulla and midbrain. There were additional small clusters (<5 voxels) of significant activation throughout the brain (see Supplementary Material Table 2 and Figure 1 for complete activation map). Only parts of this normal network were commonly activated by neurologically intact controls and patients of interest, including bilateral pre/post-central gyrus (BA 3, 4), bilateral STG (BA 22, 42), bilateral cerebellar lobule V/VI and bilateral medulla (see Supplementary Material Figure 1).

**Table 2.** Activation among neurologically intact controls.

| Region | Hemisphere | MNI Coordinate | | | Z-score | Cluster size |
| --- | --- | --- | --- | --- | --- | --- |
|  |  | **x** | **y** | **z** |  |  |
| pre/post-central gyrus (BA 3, 4) / STG (BA 22, 42) | L | -42 | -10 | 38 | Inf | 553 |
|  |  | -54 | -19 | 5 | 7.76 |  |
|  |  | -63 | -31 | 8 | 7.56 |  |
|  | R | 45 | -25 | 8 | Inf | 743 |
|  |  | 48 | -7 | 35 | Inf |  |
|  |  | 60 | -10 | -1 | Inf |  |
| pre-SMA | L | -9 | -1 | 62 | 5.58 | 10 |
|  | R | 6 | 2 | 65 | 6.48 | 25 |
| STG anterior | L | -60 | 5 | -4 | 5.36 | 6 |
| Thalamus | R | 21 | -7 | 14 | 5.36 | 11 |
| Cerebellum (lobule V/VI) | L | -21 | -58 | -22 | Inf | 156 |
|  | R | 21 | -58 | -22 | Inf | 184 |
| Cerebellum - posterior (Crus II / VIIb / VIIIa) | L | -21 | -64 | -43 | 6.38 | 23 |
|  | R | 18 | -73 | -43 | 6.88 | 111 |
| Medulla | L/R | -3 | -40 | -46 | 7.21 | 54 |
|  |  | 6 | -37 | -43 | 7.11 |  |
| Midbrain | R | 12 | -19 | -13 | 6.20 | 17 |
|  | R | 6 | -34 | -22 | 5.24 | 8 |

Main effect of 8 speech production tasks, p < 0.05 FWE-corrected across the whole brain. Regions: BA = Brodmann Area, SMA = supplementary motor area, STG = superior temporal gyrus. Hemisphere = L (left) / R (right).

**Figure 1.** Activation during speech production tasks.

**
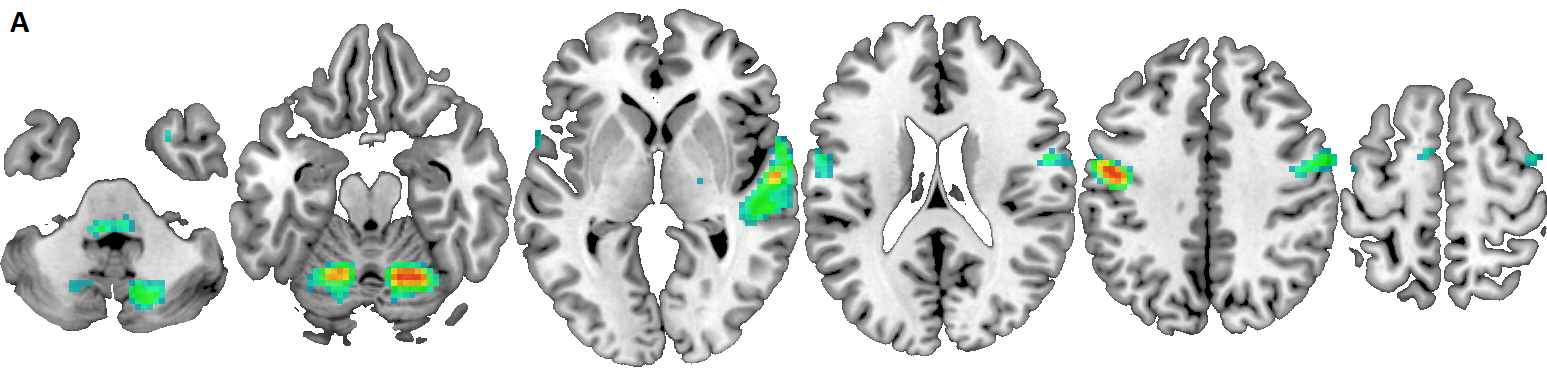

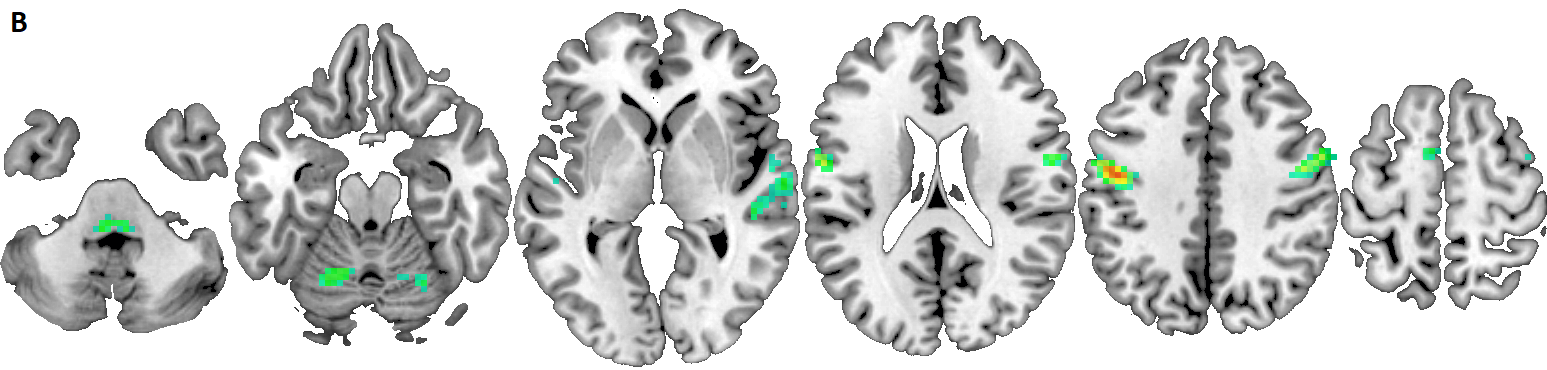
**


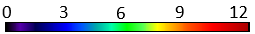


Activation during 8 speech production tasks (p < 0.05, FWE-corrected across the whole brain) in: (A) neurologically intact controls, and (B) both patients of interest and neurologically intact controls (null conjunction; Nichols *et al.*, 2005). z coordinates from left to right: -40; -20; 0; 20; 40; 60. Colour bar shows T-values.

# Speech and language abilities of patients

**Table 3.** Patient controls’ speech abilities and therapy post-stroke

| **L Cerebrum / R Cerebellum lesions** | | | | | |
| --- | --- | --- | --- | --- | --- |
| **Patient ID** | **Acute** | | **Chronic** | | **SLT** |
|  | **Self-rating** | **Notes** | **Self-rating** | **Notes** |  |
| PS0451 | Using 1 or 2 single words | Knew what they wanted to say, but words did not come out | Short sentences to normal sentences | Not quite back to normal | 50 – 100h (incl. other therapy), sorting word lists, writing |
| PS2879 | Short sentences | - | Normal sentences | Speech has greatly improved but has a faint 'drunken' slur Almost back to normal, occasional subtle difficulties when tired | 0 – 20h, Focus on word finding, slurred speech, word lists, fluency |
| PS2884 | Short sentences | Ongoing expressive speech difficulties (speech dysfluency), which is improving since stroke | Short to normal sentences | Speech becomes stilted in demanding situations, neurogenic stammer which was still audible | < 20h, word fluency, semantic association, motor exercises |
| PS3021 | Using 1 or 2 single words | Only able to say words yes/no.  Later: production of simple sentences | Short sentences | Slight stammer | - |
| PS3476 | Using gestures but not speaking at all | Volume was very quiet, vocal weakness / huskyness (lessened over months) | Short to normal sentences | Rate of speech is slower, difficulty speaking in a group | > 20h, voice work: projecting voice better |
| PS3488 | Short sentences | Could only say “oh my god” | Short to normal sentences | Speech took some time to recover, rates speech now at ~60% compared to pre-stroke | < 20h, facial muscle exercises |
| PS2386 | Short sentences | Mumbles/blurs words when tired or stressed,  slurred speech,  speech problems continue to minor extent | Normal sentences | Speech recovered by ~9 months but it was a slow progression | < 20h, forming words (naming, reading, repeating), oro-motor exercises |
| PS1627 | Short to normal sentences | Could not say much because of weakness/processing | Short to normal sentences | Occasional stuttering/mumbling | < 20 h, reading, talking about events |
| PS0369 | Not speaking at all nor using gestures | Could only make noises & trying to use gestures unsuccessfully | Short to normal sentences | Difficulties when tired | < 20h |
| PS1343 | Short sentences | - | Short to normal sentences | - | < 20h, Comprehension |

| **R Cerebrum / L Cerebellum lesions** | | | | | |
| --- | --- | --- | --- | --- | --- |
| **Patient ID** | **Acute** | | **Chronic** | | **SLT** |
|  | **Self-rating** | **Notes** | **Self-rating** | **Notes** |  |
| PS2003 | Using gestures but not speaking at all | - | Short sentences | - | < 20h, repetition, use of voice/quality of voice |
| PS2797 | Normal sentences | Slurred speech, lasted ~ 1 day Pausing between words | Normal sentences | - | - |
| PS2872 | Normal sentences | 'Overly' answers questions, trouble stopping speech | Normal sentences | Speech problems have improved, still unable to stop self-speaking at times | - |
| PS2981 | Normal sentences | No speech in the first week due to fatigue | Normal sentences | - | < 20 h, activities similar to CAT |
| PS3189 | Normal sentences | Dysphagia, prolapse left mouth corner | Normal sentences | No problems with speech | - |
| PS3235 | Normal sentences | - | Normal sentences | - | - |
| PS3608 | Normal sentences | Speech unaffected | Normal sentences | - | - |
| PS0472 | Short sentences | Talking 'gibberish' | Short to normal sentences | Speaking got worse as the day went on at work, still improving | - |

| **Bilateral cerebrum lesions** | | | | | |
| --- | --- | --- | --- | --- | --- |
| **Patient ID** | **Acute** | | **Chronic** | | **SLT** |
|  | **Self-rating** | **Notes** | **Self-rating** | **Notes** |  |
| PS0673 | Using 1 or 2 single words | Some automatic swearing once started to speak  resolved in 3 months | Short sentences | Initial severe expressive aphasia, Speech resolved in 3 years | 20 – 50h, categorisation tasks, remembering words, group work |
| PS3315 | Using gestures but not speaking at all | Could not speak for 6 weeks post stroke | Short sentences | - | ̴6 weeks |

“-“ = no notes describing speech abilities / SLT; Acute = 1 month post-stroke; Chronic = at time of testing; SLT = Speech and Language Therapy. Self-rating: patients retrospectively evaluated their speech abilities, indicating on a scale whether they were unable to attempt speech, speak or use gestures; or able to use gestures but not speak; use only 1 or 2 single words; use a few single words; speak in short sentences; or speak normally.

**Table 4**. Number and type of errors produced during single word production.

| **Patient ID** | **Naming and Repetition**  **(53 items)** | | | **Spoken Picture Description** | | | **fMRI**  **(80 items)** | | |
| --- | --- | --- | --- | --- | --- | --- | --- | --- | --- |
|  | **Language** | | **Articulation** | **Language** | | **Articulation** | **Language** | | **Articulation** |
|  | **n** | **Type** | **n** | **n** | **Type** | **n** | **n** | **Type** | **n** |
| **Patients of interest** | | | | | | | | | |
| PS2068 | 2 | 1 sem  1 phon | 2 | 2 | sem | 0 | 1 | sem | 0 |
| PS2464 | 2 | sem | 2 | 1 | sem | 0 | 3 | 2 pho  1 NR | 1 |
| PS1327 | 0 | - | 0 | 0 | - | 0 | 3 | 1 phon  2 sem | 5  1 (phon/art) |
| PS2504 | 0 | - | 0 | 2 | sem | 1 | 0 | - | 0 |
| PS0573 | 1 | unknown | unknown | 0 | - | 0 | 0 | - | 0 |
| PS1575 | 2 | reg | 0 | 1 | sem | 0 | 3 | 2 sem  1 NR | 0 |
| PS0452 | 2 | unknown | unknown | 0 | - | 0 | 4 | 3 NR  1 sem | 3 |
| **Patient controls; L Cerebrum and/or R Cerebellum lesions** | | | | | | | | | |
| PS0451 | 1 | unknown | unknown | 0 | - | 0 | 3 | 2 NR  1 sem | 1 |
| PS2879 | 1 | phon | 0 | 0 | - | 0 | 0 | - | 1 |
| PS2884 | 0 | - | 0 | 0 | - | 1 | 4 | 4 NR | 1 |
| PS3021 | 1 | sem | 0 | 1 | sem | 1 | 0 | - | 0 |
| PS3476 | 0 | - | 0 | 0 | - | 0 | 0 | - | 1 |
| PS3488 | 3 | 1 sem  2 gra | 0 | 2 | sem | 0 | 6 | NR | 0 |
| PS2386 | 1 | reg | 0 | 2 | sem | 0 | 10 | 1 phon  3 unc  6 NR | 4 (phon/art) |
| PS1627 | 1 | sem | 1 | 3 | sem | 0 | 1 | 1 sem | 1  1 (phon/art) |
| PS0369 | 0 | - | unknown | 1 | sem | 0 | 4 | 3 sem  1 NR | 2 |
| PS1343 | 2 | 1 phon  1 reg | 0 | 0 | - | 0 | 3 | NR | 4  1 (phon/art) |
| **Patient Controls; R Cerebrum and/or L Cerebellum lesions** | | | | | | | | | |
| PS2003 | 0 | - | 0 | 0 | - | 1 | 0 | - | 0 |
| PS2797 | 0 | - | 0 | 1 | neologism | 0 | 1 | sen | 0 |
| PS2872 | 3 | 1 sem  2 phon | 0 | 3 | sem | 0 | 0 | - | 1 |
| PS2981 | 1 | phon | 0 | 0 | - | 0 | 2 | sem | 0 |
| PS3189 | 2 | phon | 0 | 2 | sem | 0 | 3 | 1 sem  1 unc  1 NR | 0 |
| PS3235 | 2 | 1 sem  1 phon | 0 | 1 | sem | 0 | 1 | sem | 0 |
| PS3608 | 3 | phon | 0 | 0 | - | 0 | 2 | 1 unc  1 phon | 0 |
| PS0472 | 0 | - | unknown | 0 | - | 0 | 0 | - | 0 |
| **Patient Controls; Bilateral cerebrum lesions** | | | | | | | | | |
| PS0673 | 0 | - | unknown | 0 | - | 1 | 3 | 2 sem  1 NR | 3 |
| PS3315 | 2 | phon | 0 | 0 | - | 0 | 2 | gra | 0 |

Naming, Repetition and Spoken Picture Description tasks are taken from the Comprehensive Aphasia Test (CAT). For some patients there was no recording of the Naming and Repetition tasks from the CAT, therefore we could not score articulation errors. This is marked as ‘unknown’. From the fMRI tasks we coded errors for the visual object naming (vO) and auditory word repetition (aW), with each task having 40 items.

Errors classification:

(i) Phonological (phon; e.g. repeating non-word ‘spenk’ as ‘spink’)

(ii) Semantic (sem; naming a picture of ‘whale’ as ‘fish’)

(iii) Grammar (gra; e.g. adding ‘s’ to a picture of singular item)

(iv) Regularisation (reg; e.g. repeating non-word ‘spenk’ as ‘spent’)

(v) No response (NR)

(vi) Neologism (e.g. describing a shelf under a coffee table as an ‘under-stand’)

(vii) Unclassified (unc; e.g. repeating ‘sofa’ as ‘scooter’; naming a picture of ‘fridge’ as ‘book’)

(viii) Articulation errors include dyspraxic and dysarthric errors (e.g. repeating ‘frog’ as ‘fwog’). In cases where it was unclear whether the error is of phonetic or articulatory origin, we classified it as either (‘phon/art’), and listed it as an articulatory error in the table above, in order to avoid under-estimation of patients’ articulatory deficits.

# fMRI tasks performance

**Table 5**. Performance on the fMRI tasks

(A) Accuracy (percent correct)

| **ID / Group** | **vW** | **vP** | **vO** | **vC** | **aW** | **aP** | **aO** | **aH** |
| --- | --- | --- | --- | --- | --- | --- | --- | --- |
| **POI** |  |  |  |  |  |  |  |  |
| PS2068 | 100 | 98 | 98 | 95 | 100 | 98 | 88 | 100 |
| PS2464 | 100 | 100 | 100 | 100 | 90 | 93 | 73 | 100 |
| PS1327 | 100 | 100 | 95 | 100 | 100 | 98 | 95 | 100 |
| PS2504 | 100 | 93 | 100 | 88 | 100 | 95 | 80 | 90 |
| PS0573 | 100 | 98 | 98 | 100 | 100 | 100 | 93 | 95 |
| PS1575 | 98 | 83 | 90 | 88 | 100 | 93 | 70 | 93 |
| PS0452 | 98 | 93 | 93 | 93 | 90 | 85 | 63 | 93 |
| Mean | 99 | 95 | 96 | 95 | 97 | 94 | 80 | 96 |
| SD | 1.2 | 6.2 | 3.8 | 5.7 | 4.9 | 4.9 | 12.2 | 4.3 |
| **PC** |  |  |  |  |  |  |  |  |
| Mean | 98 | 95 | 94 | 99 | 95 | 91 | 81 | 93 |
| SD | 5.7 | 7.8 | 5.2 | 2.5 | 9 | 9.8 | 9.8 | 8.9 |
| Min | 75 | 75 | 80 | 93 | 63 | 65 | 63 | 68 |
| Max | 100 | 100 | 100 | 100 | 100 | 100 | 98 | 100 |
| **NC** |  |  |  |  |  |  |  |  |
| Mean | 100 | 97 | 95 | 99 | 99 | 96 | 89 | 97 |
| SD | 0.9 | 4.7 | 5.4 | 1.1 | 1.6 | 4.5 | 11.9 | 5.9 |
| Min | 98 | 88 | 80 | 98 | 95 | 85 | 65 | 78 |
| Max | 100 | 100 | 100 | 100 | 100 | 100 | 100 | 100 |

fMRI Tasks: word reading (vW); pseudoword reading (vP); object naming (vO); colour naming (vC); word repetition (aW); pseudoword repetition (aP); auditory object naming (aO); naming gender of humming voice (aH). POI: patients of interest; PC: patient controls; NC: neurologically intact controls; SD: standard deviation

(B) Response Time (msec)

| **ID / Group** | **vW** | **vP** | **vO** | **vC** | **aW** | **aP** | **aO** | **aH** |
| --- | --- | --- | --- | --- | --- | --- | --- | --- |
| **POI** |  |  |  |  |  |  |  |  |
| PS2068 | 881 | 1135 | 1072 | 999 | 1173 | 1182 | 1452 | 1294 |
| PS2464 | - | 762 | 1279 | 913 | 1417 | 1263 | 1752 | 1772 |
| PS1327 | 819 | 865 | 1128 | 1034 | 1243 | 1269 | 1591 | 1620 |
| PS2504 | 907 | 1199 | 1166 | 1039 | 1193 | 1297 | 1714 | 1442 |
| PS0573 | 792 | 949 | 1031 | 881 | 1180 | 1137 | 1387 | 1470 |
| PS1575 | 864 | 1006 | 953 | 822 | 1107 | 1164 | 1438 | 1304 |
| PS0452 | 1096 | 1290 | 1404 | 1344 | 1207 | - | 1680 | 1456 |
| Mean | 893 | 1029 | 1148 | 1005 | 1217 | 1219 | 1573 | 1480 |
| SD | 107.5 | 188.7 | 153.3 | 170.6 | 97.2 | 65.9 | 148.1 | 169.4 |
| **PC** |  |  |  |  |  |  |  |  |
| Mean | 979 | 1105 | 1210 | 1021 | 1371 | 1419 | 1731 | 1615 |
| SD | 209.6 | 182.2 | 132.5 | 185 | 124.5 | 132.3 | 183.5 | 225.2 |
| Min | 690 | 856 | 1004 | 702 | 1141 | 1199 | 1407 | 1118 |
| Max | 1421 | 1517 | 1461 | 1282 | 1592 | 1689 | 2067 | 2012 |
| **NC** |  |  |  |  |  |  |  |  |
| Mean | 811 | 970 | 1060 | 896 | 1223 | 1319 | 1601 | 1455 |
| SD | 98.9 | 115.5 | 121.9 | 95.6 | 104.6 | 109.5 | 232.4 | 200 |
| Min | 701 | 788 | 859 | 768 | 1116 | 1162 | 1150 | 1091 |
| Max | 959 | 1155 | 1281 | 1058 | 1472 | 1483 | 1894 | 1872 |

fMRI Tasks: word reading (vW); pseudoword reading (vP); object naming (vO); colour naming (vC); word repetition (aW); pseudoword repetition (aP); auditory object naming (aO); naming gender of humming voice (aH). POI: patients of interest; PC: patient controls; NC: neurologically intact controls; SD: standard deviation

# Enhanced r-PMd and r-SMA activation in patient controls

Activation that was higher than maximum activation in neurologically intact controls was not exclusive to our patients with focal cerebellar lesions, as it was also observed in:

1. Both r-PMd and r-SMA, for two patient controls with damage to the left sub-insula and putamen (PS3476 and PS3021).
2. r-PMd not r-SMA, in a patient with left subcortical damage deep to the insula (PS0451), and a patient with large right MCA lesions (PS3235).
3. r-SMA not r-PMd, in a patient with damage to the left ventral cerebellum Crus II (PS0472), and two patients with a large right hemisphere lesion that damaged temporal and ventral frontal regions, insula and basal ganglia (PS2872 and PS2981).

# Inter-patient variability in activation among patients of interest

**Figure 2**. The (non-significant) relationships between activation and other variables.


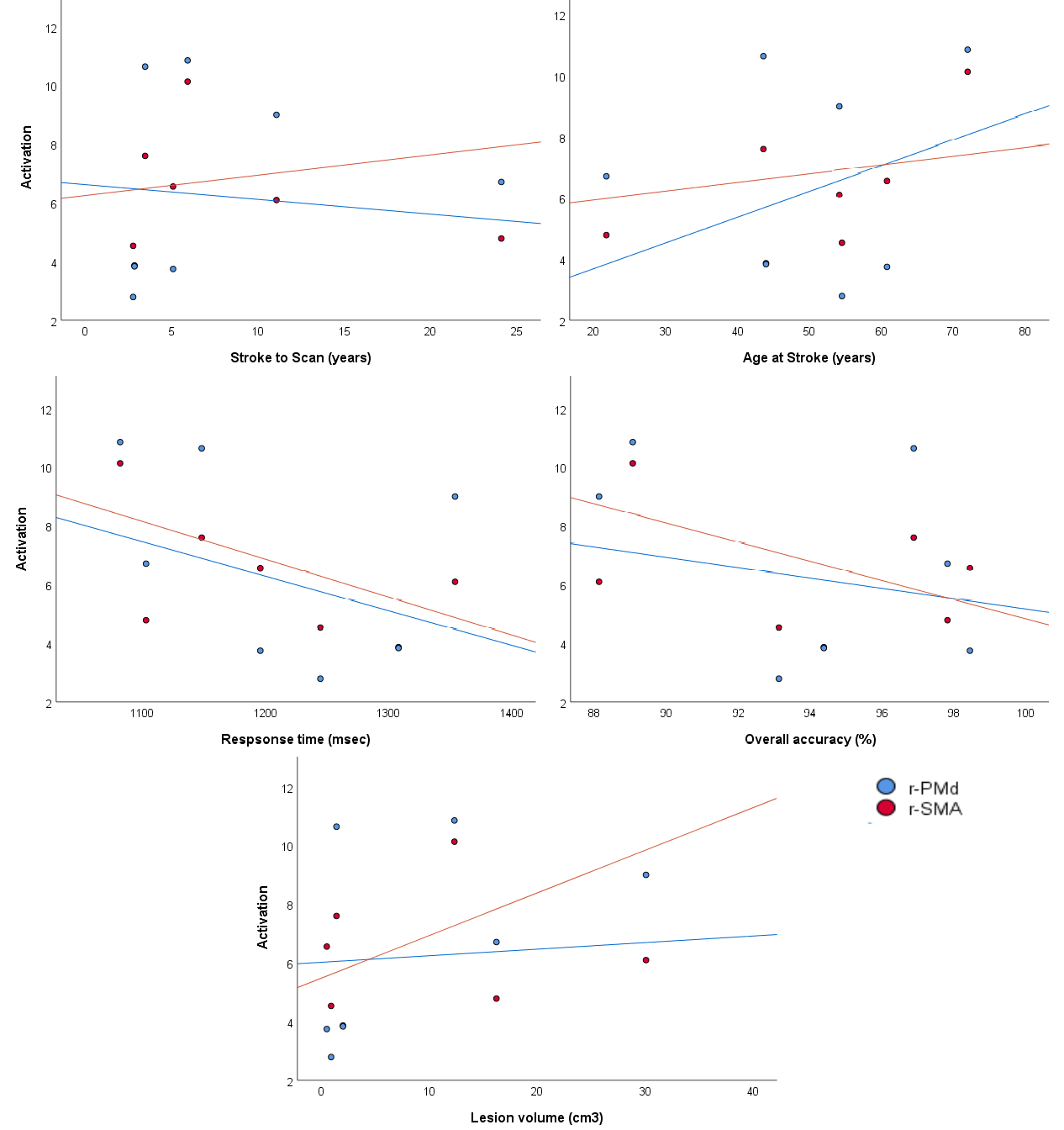


Y axis represents the principal eigenvariate extracted from 3 mm radius spheres centred on the peak coordinates reported in Table 4, in the right dorsal premotor cortex (r-PMd, Blue) and right supplementary motor area (r-SMA, Red).
